# Identifying MicroRNAs Involved in Degeneration of the Organ of Corti during Age-Related Hearing Loss

**DOI:** 10.1371/journal.pone.0062786

**Published:** 2013-04-30

**Authors:** Qian Zhang, Huizhan Liu, JoAnn McGee, Edward J. Walsh, Garrett A. Soukup, David Z. Z. He

**Affiliations:** 1 From the Department of Biomedical Sciences, Creighton University School of Medicine, Omaha, Nebraska, United States of America; 2 Boys Town National Research Hospital, Omaha, Nebraska, United States of America; Wayne State University, United States of America

## Abstract

MicroRNAs (miRNAs), a class of short non-coding RNAs that regulate the expression of mRNA targets, are important regulators of cellular senescence and aging. We questioned which miRNAs are involved in age-related degeneration of the organ of Corti (OC), the auditory sensory epithelium that transduces mechanical stimuli to electrical activity in the inner ear. Degeneration of the OC is generally accepted as the main cause of age-related hearing loss (ARHL), a progressive loss of hearing in individuals as they grow older. To determine which miRNAs are involved in the onset and progression of ARHL, miRNA gene expression in the OC of two mouse strains, C57BL/6J and CBA/J, was compared at three different ages using GeneChip miRNA microarray and was validated by real-time PCR. We showed that 111 and 71 miRNAs exhibited differential expression in the C57 and CBA mice, respectively, and that downregulated miRNAs substantially outnumbered upregulated miRNAs during aging. miRNAs that had approximately 2-fold upregulation included members of miR-29 family and miR-34 family, which are known regulators of pro-apoptotic pathways. In contrast, miRNAs that were downregulated by about 2-fold were members of the miR-181 family and miR-183 family, which are known to be important for proliferation and differentiation, respectively. The shift of miRNA expression favoring apoptosis occurred earlier than detectable hearing threshold elevation and hair cell loss. Our study suggests that changes in miRNA expression precede morphological and functional changes, and that upregulation of pro-apoptotic miRNAs and downregulation of miRNAs promoting proliferation and differentiation are both involved in age-related degeneration of the OC.

## Introduction

Age-related hearing loss (ARHL), also known as presbycusis, is a progressive sensorineural hearing loss that occurs as people get older. It has been reported that as many as 35% to 50% of the population aged between 65 and 75 have ARHL [1], [2]. Although gradual morphological and physiological changes in the central auditory system can also contribute to hearing impairment and difficulty understanding spoken language, it is generally accepted that degeneration of the organ of Corti (OC) is the primary cause of ARHL. The OC contains mechanosensitive hair cells, which convert mechanical stimuli into electrical activity.

miRNAs are endogenous, small (20–23 nt), non-coding RNAs that bind to complementary sequences within target messenger RNA (mRNA) transcripts and typically result in translational repression or target degradation and gene silencing [3]. miRNAs are collectively predicted to target ∼60% of all genes and each miRNA is expected to repress hundreds of target genes. miRNAs are a vital part of genetic regulation and exhibit a wide range of biological functions including cell differentiation, proliferation, apoptosis, metabolism, and self-renewal [4]. Recent studies have established a direct correlation between miRNA regulation and aging in worms (*Caenorhabditis elegans*), mice, and humans [5], [6]. Lin-4 is the first miRNA identified that is highly related to life span in *C. elegans* [3], [7]. A number of other miRNAs including the miR-29 family, miR-34 family, miR-15/16, miR-17-92 cluster, miR-146a/b, and miR-200 family are all known to be involved in networks regulating cell senescence and death [8]–[10].

Approximately one-fourth of known mouse miRNAs are expressed in the inner ear of mice [11]–[13]. Many of these miRNAs are associated with proliferation, differentiation, and morphological/functional development of auditory sensory epithelia [14]–[16]. For example, miR-124 and the miR-183 family are necessary for neurosensory cell fate determination [14], [15], [17]. Other studies have shown that miR-181a and the let-7 family play important roles in hair cell regeneration in chicken and newt [18]–[20]. Although miRNAs are known to affect cellular proliferation, differentiation, and growth in the inner ear, the role of miRNAs in aging of the OC has not been examined. The goal of our study was to identify miRNAs that were differentially expressed in the OC between younger and older mice. Identification of which miRNAs are indicated in aging is a first step in the effort to elucidate the roles of miRNAs and their regulatory networks in age-related degeneration of the OC.

We reasoned that miRNAs that are involved in the age-related degeneration of the OC should be differentially expressed before and after the onset of ARHL. GeneChip miRNA microarray was used to examine miRNA expression in the OC at three different ages in two mouse strains, C57BL/6J and CBA/J. We then used quantitative real-time PCR (q-PCR) to validate the expression profiles of four individual miRNAs that were indicated by microarray analysis. To compare and correlate differential miRNA expression profiles with functional and morphological changes of hair cells, we also examined hearing thresholds and hair cell morphology at different stages before and after the onset of ARHL.

## Materials and Methods

### OC Tissue Collection

C57BL/6J and CBA/J mice were used for the study. Both strains were bred in-house after purchase from Jackson Laboratory (Bar Harbor, ME, USA). Care and use of the animals in this study were approved by grants from the National Institutes of Health and by the Institutional Animal Care and Use Committees of Creighton University and Boys Town National Research Hospital.

Cochleae were rapidly dissected in cold phosphate-buffered saline (PBS; 10 mM Na_2_HPO_4_, 1.7 mM KH_2_PO_4_, 137 mM NaCl, 2.7 mM KCl, pH7.4) after the mice were euthanized. The basilar membrane together with the OC was isolated. Tissue from ten cochleae from five mice was pooled as one group for each GeneChip microarray analysis. Three groups for each age and mouse strain were prepared using C57 mice at postnatal day 21 (P21), 3 months (3 m) and 9 months (9 m), and CBA mice at P21, 9 m, and 16 months (16 m). Isolated tissues were stored at −20°C in RNAlater stabilization reagent (Ambion, Austin, TX, USA). Total RNA including miRNAs was isolated using the mirVana miRNA Isolation Kit (Ambion) and dissolved into 20–30 µl of RNase-free water. RNA concentration was determined by UV spectrophotometry (Nanodrop ND-1000), and RNA quality was examined by measuring the ratio of 28S to 18S rRNA using an Agilent 2100 BioAnalyzer.

### GeneChip Microarray of miRNAs

The miRNA gene expression profile of each OC tissue sample was determined by GeneChip microarray analysis (Affymetrix, Santa Clare, CA, USA) using approximately 250 ng of total RNA obtained from each group. Synthesis of cDNA, hybridization to chips, and washes were performed according to the manufacture’s protocol. GeneChips were scanned at 3 µm density with a GeneArray Scanner (Affymetrix). Images were inspected to ensure that all chips had low background but bright hybridization signals. Mean fluorescence signal intensity for each probe was quartile normalized. The average of three mean signals for each miRNA probe was normalized to that for an added control oligonucleotide and was log2 transformed. Each miRNA probe was assessed for expression based on a Wilcoxon Rank-Sum test of the miRNA probe set signals compared to the distribution of signals from the background. The Student’s t-test was used to determine significant differences in miRNA expression between P21 and older-aged groups, where p<0.05 was interpreted as significant.

### Quantitative Real-time PCR

For quantitative real-time PCR (q-PCR) analysis of miRNAs, the apical turn of the cochlea from five additional animals for each group from different ages of C57 and CBA mice was obtained. q-PCR detection of miRNAs was performed using mirVana q-PCR miRNA Primer sets (Ambion). 100 ng total RNA from each group was reverse transcribed using SuperScript Reverse Transcriptase (Invitrogen) in a 20 µl reaction to produce cDNA. q-PCR was performed using a 7500 Fast Real-Time PCR System (Applied Biosystems) to analyze triplicate reactions (20 µl) containing 2× SYBR Green PCR Master Mix (Applied Biosystems), a 10-fold dilution of mirVana q-PCR Primer Set, and cDNA. After incubation at 95°C for 20 seconds, PCR products were analyzed throughout 40 cycles consisting of an incubation at 95°C for 3 s and 60°C for 30 s. U6 RNA was detected as an internal relative control for each group. The threshold cycle (C_T_) was defined as the PCR cycle number at which the fluorescence intensity was appreciably above the background level but was still in the early exponential phase of amplification. The change in C_T_ between a given miRNA and U6 RNA for each reaction was defined as ΔC_T_ and averaged for each group. The change in ΔC_T_ between two groups was defined as ΔΔC_T_, which represented a relative difference in expression of miRNA. Fold difference in miRNA expression between groups was calculated as 2^−ΔΔCT^. We compared the ΔCT values for each miRNA from older groups with those for the P21 group using the Student’s t-test, where p<0.05 was interpreted as significant.

### Hearing Threshold Measurement Using Auditory Brainstem Responses (ABR)

Five animals for each mouse strain were used for ABR recordings. The mouse was anesthetized with mixture of ketamine/xylazine (ketamine 100 mg/kg; xylazine 15 mg/kg; ip), and supplemented as needed. ABRs were recorded in response to tone bursts of 2, 2.8, 4, 5.6, 8, 11, 16, 22, 32, 40, 50, and 60 kHz using standard procedures previously described [21]. Tone bursts with 1 ms rise cosine on/off ramps were generated digitally using a clock rate of 125 kHz and 16-bit D/A converters. Stimulus levels were calibrated using a 1/8″ Brüel and Kjær microphone (Model 4138) and were presented in sound pressure level in decibel (dB SPL: referenced to 20 µPa). ABR signals were collected with subcutaneous platinum needle electrodes placed at the vertex, mastoid prominence, and shoulder. Response signals were amplified (100,000×), filtered, and acquired by TDT Workstations (Tucker-Davis Technologies). Each averaged response was based on 200 stimulus repetitions. During the procedure, the body temperature was maintained at 38°C with a heating pad. All records were obtained in a sound-attenuating chamber.

### Hair Cell Counting

The cochleae of CBA (at the age of P21, 9 m, and 16 m) and C57 mice (at P21, 3 m, and 9 m) were perfused with 4% paraformaldehyde (PFA) after transcardiac perfusion and maintained in the fixative at 4°C overnight. The basilar member including the OC was dissected and cut into two (apical and basal) segments. The tissue was blocked for 1 hour with 0.25% normal goat serum in PBS containing 0.01% Triton-X-100 for 1 hour. Primary antibody for Myo VIIa (Invitrogen) was diluted 1∶200 and incubated for 24 hours at 4°C. After several washes with PBS, secondary antibody (1∶500) (Alexa fluor molecular probe 488; Invitrogen) was added and incubated overnight at 4°C. Tissues were washed with PBS and mounted on slides with antifade solution (5-ml PBS, 5-ml glycerol, 0.1-g n-propyl gallate). Images were captured using a LSM 510 META confocal scanning system with three lasers mounted on a Zeiss AxioPlan 2IE MOT motorized upright microscope (Carl Zeiss International). Hair cell counts from two areas (400 µm in length) at the basal and apical turns were obtained from confocal images offline.

## Results

### 1.Hearing Thresholds of C57 and CBA Mice

**Figure 1:**
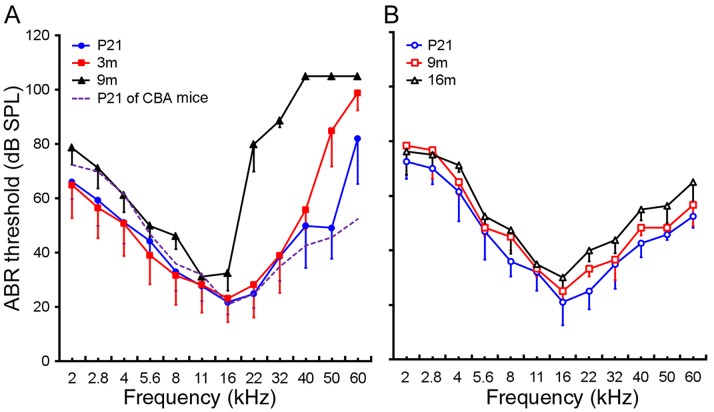
ABR thresholds of C57 and CBA mice. **A**: ABR thresholds of C57 mice at the ages of P21, 3 m, and 9 m. The dotted lines are the ABR threshold of CBA mice at P21. **B**: ABR thresholds of CBA mice at the ages of P21, 9 m, and 16 m.

The CBA and C57 strains are the most widely used mouse models of ARHL [22], [23]. The CBA strain has a slow, progressive hearing loss that occurs near 18 months after birth [22]–[26]. The C57 strain possesses a mutation in the *Ahl* gene that leads to deficiencies in the cadherin 23 protein [27], [28], an important component of the transduction apparatus in the stereocilia of hair cells [29]–[31]. The C57 mice have an accelerated ARHL, as reflected in rapid deterioration in ABR thresholds and declines in distortion product otoacoustic emission levels that start in the higher frequencies (basal turn) and progress to lower frequencies (apical turn) with aging [24], [32], [33]. We measured ABR-based hearing thresholds of C57 and CBA mice to determine when hearing loss began in these two strains. Although ABR thresholds of these two strains were previously examined [22], [24], [26], we used a wider range of frequencies to determine thresholds at higher frequencies. ABR thresholds were measured at the ages of P21, 3 m, and 9 m for C57 mice, and at P21, 9 m, and 16 m for CBA mice. Five animals from each strain were used. Figure 1 shows the thresholds (presented as means ± sd) obtained from C57 and CBA mice using tone bursts with frequencies varied from 2 to 60 kHz. For comparison, thresholds of CBA mice at P21 are also presented in Figure 1A, along with the audiograms of C57 mice. C57 and CBA mice showed some difference at the low and high frequency ends. C57 mice had a threshold elevation of approximately 30 dB at the frequency of 60 kHz at P21 compared to the threshold of CBA mice at the same age (Fig. 1A). At the age of 3 m, C57 mice exhibited a significant elevation of the threshold at 50 and 60 kHz. Hearing loss extended toward middle and low frequencies during aging, and by 9 m hearing loss greater than 50 dB was observed at 22 kHz and greater. In contrast, the ABR thresholds of CBA mice remained unchanged between P21 and 16 m (Fig. 1B). The ABR audiogram indicates that C57 mice displayed an onset and rapid progression of hearing loss starting from high frequencies as early as 3 months after birth, whereas CBA mice exhibit minimal hearing loss even at 16 m.

### 2.Hair Cell Counting

**Figure 2:**
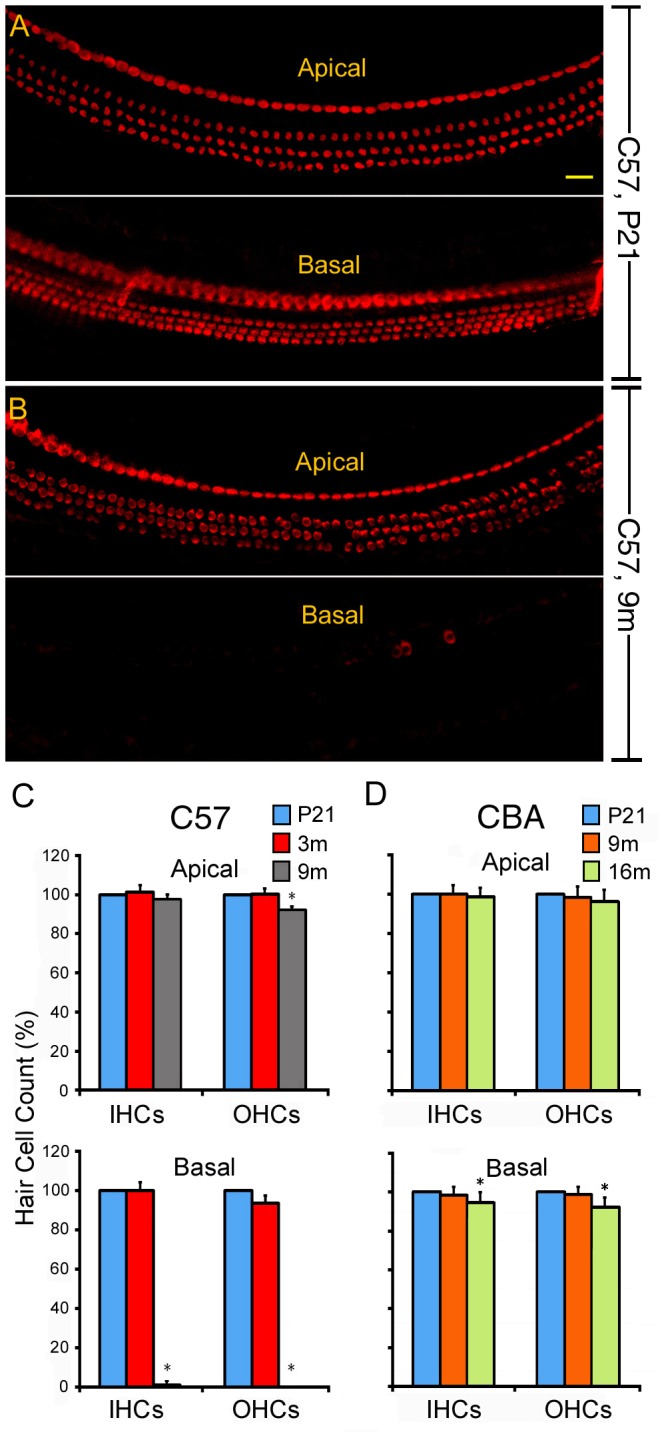
Hair cell counts from the OC of C57 and CBA mice. **A, B:** Representative confocal images of myo7a-labeled hair cells at the apical and basal turns of C57 mice at P21 (A) and 9 m (B). **C, D:** Hair cell counts obtained from two representative cochlear locations from C57 (C) and CBA (D) mice at different ages. The two locations were approximately 0.5–0.9 and 5.0–5.4 mm from the basal end of the basilar membrane. IHCs and OHCs were counted from each location. Three animals for each strain and age were included in the counts. The average numbers of IHCs and OHCs at each location are normalized to those of P21 mice (100%). Asterisks indicate statistically significant differences (p<0.05, Student’s t-test) compared to P21.

We examined hair cell loss at two cochlear locations at different ages for the two mouse strains. The total length of the basilar membrane of the mouse cochlea is approximately 5.8 mm (measured from middle point of the basilar membrane) based on a study by Müller et al. [34]. The locations where hair cell count were taken were approximately 0.5–0.9 and 5.0–5.4 mm from the basal end of the basilar membrane, representing two regions in the basal and apical turns with best frequencies of 55–45 and 2–3 kHz, respectively [34]. As shown in Figure 2A, there was no apparent hair cell loss in this near-hook region in the basal turn at P21 for C57 mice, despite a 30-dB deficit compared to the threshold of CBA mice at 60 kHz. Thus, the threshold elevation might be either inherent to C57 mice or due to ultrastructural changes in the hair cells that could not be detected upon gross morphological examination. At the age of 3 m, sporadic outer hair cell loss was observed in the basal region of the C57 mice. However, no significant hair cell loss was detected in the apical region. By 9 m, a complete loss of outer hair cells and 96% loss of inner hair cells were observed in the basal turn region (Fig. 2B and 2C). In the apical turn region, there was less than 10% loss of outer hair cells and no significant loss of inner hair cells. For CBA mice, no significant hair cell loss was detected in either of the two regions at the age of 9 m (Fig. 2D). At 16 m, the apical turn region exhibited no significant hair cell loss, whereas a 5% loss of inner hair cells and an 8% loss of outer hair cells were observed in the basal region. Thus, hearing loss and hair cell loss observed in 3-month-old C57 mice were greater than those of 16-month-old CBA mice.

### 3.Differential Expression of miRNAs in the OC during Aging

**Figure 3:**
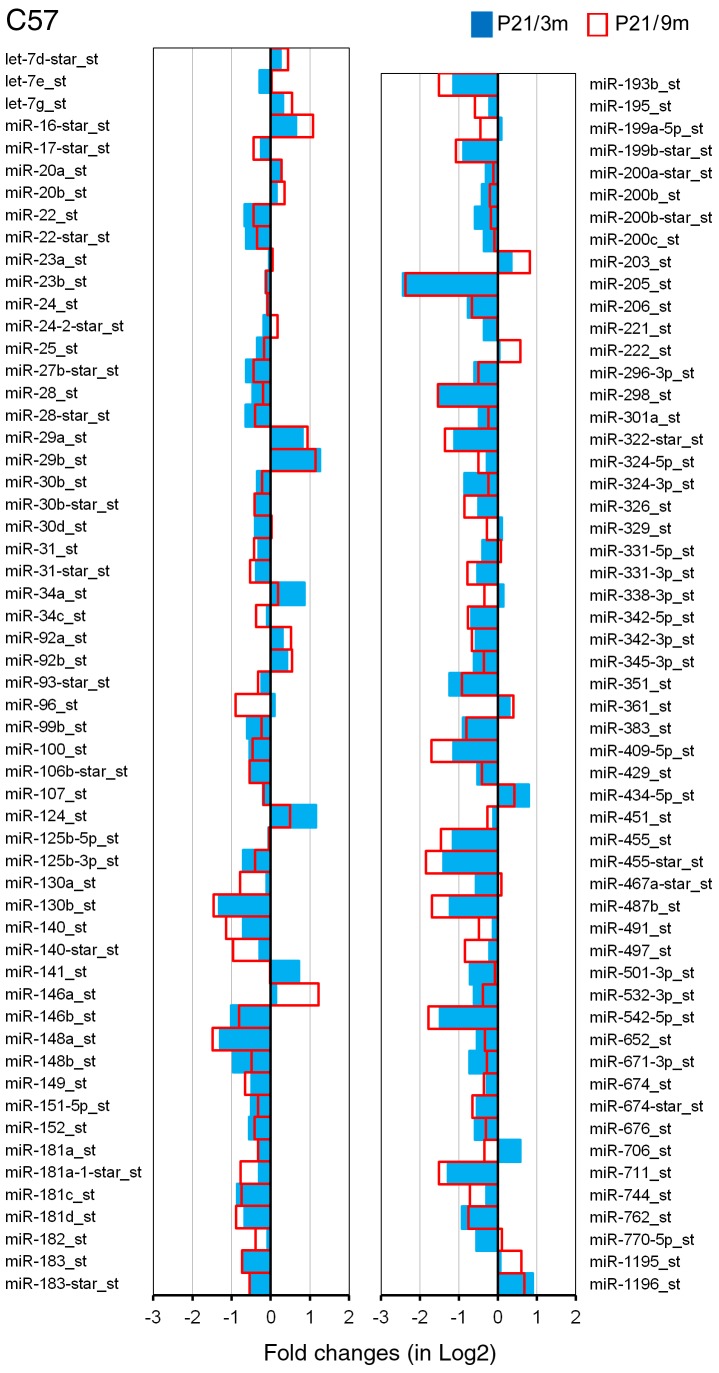
Differentially expressed miRNAs in the OC of C57 mice during aging. The X-axis relates Log2 transformed fold change in miRNA expression at 3 m (in blue) and 9 m (in red) compared to P21. Only those miRNAs which showed a statistically significant different in expression (p<0.05, Student’s t-test) compared to P21 are shown.

**Figure 4:**
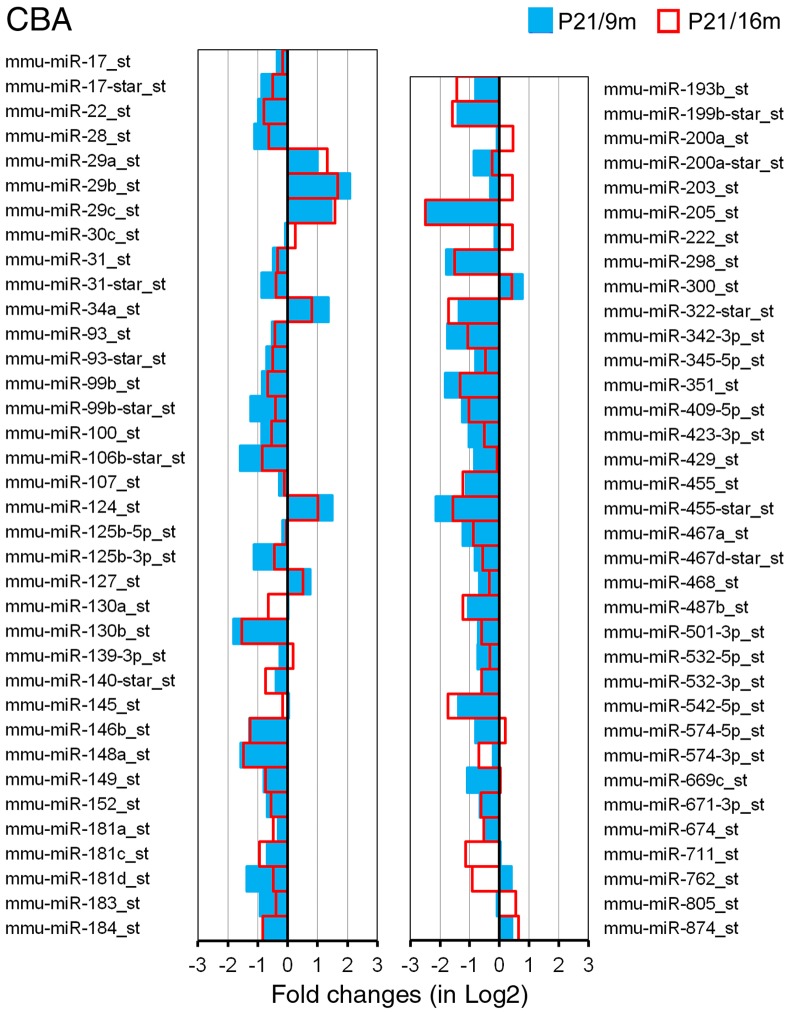
Differentially expressed miRNAs in the OC of CBA mice during aging. The X-axis relates Log2 transformed fold change in miRNA expression at 9 m (in blue) and 16 m (in red) compared to P21. Only those miRNAs which showed a statistically significant different in expression (p<0.05, Student’s t-test) compared to P21 are shown.

The expression profiles of miRNAs presented in this study represented 3 repeats for each age/species for the GeneChip microarray. The raw data of miRNA array can be downloaded from the National Center for Biotechnology Information-Gene Expression Omnibus (GEO) (GEO submissions number: GSE45026). A total of 45,930 miRNA genes were probed in the OC tissues, including 7788 probe sets that covered a variety of other species. Our analyses were performed on all known mouse miRNAs (mmu-miRNAs). There were 609 probes of mmu-miRNAs. Two criteria were used for identifying differentially expressed miRNAs between younger and older mice: first, the miRNA had to be “expressed” in each of the two samples for each age group; second, the expression level of the miRNAs had to be significantly different from that of the younger group (p<0.05, student’s t-test). miRNAs that met the two criteria are presented in Figures 3 and 4.

P21 mice were selected as reference for screening miRNAs that were differentially expressed during aging for both strains. At this age, the OC is known to be structurally and functionally adult-like while no hair cell loss is detected. We examined miRNA differential expression between P21 and 3 m as well as between P21 and 9 m in C57 mice. The selection of 3- and 9-month-old mice as the older groups for C57 mice was based on the fact that onset of high frequency hearing loss began in the basal turn at 3 m, and by 9 m hearing loss and hair cell loss were extended to mid-frequencies. Thus, these two ages represent the onset and progression of ARHL in C57 mice. Figure 3 presents differential expression profiles of miRNAs from the OC of C57 mice between P21 and 3 m (in blue), as well as between P21 and 9 m (in red). A total of 111 miRNAs were identified as significantly differentially expressed. Among them, ninety-eight (88%) miRNAs showed changes in the same direction (upregulation or downregulation) at the two different ages. miRNAs that were downregulated during aging outnumbered upregulated miRNAs by a ratio of approximately 4.4∶1 (80/18). Sixteen miRNAs had their expression level downregulated by more than 50%, whereas 10 miRNAs were upregulated by more than twofold.

ABR-based threshold measurements showed that CBA mice did not have significant high frequency hearing loss even at 16 months after birth. We compared miRNA expression of CBA mice between P21 and 9 m, as well as between P21 and 16 m. Comparison between P21 and 9 m allowed us to determine whether changes in the expression profile of miRNAs start much earlier than any morphological and functional changes are detected. As shown in Figure 4, 71 miRNAs were found to be differentially expressed. Fifty-nine (83%) miRNAs exhibited expression-level changes in the same directions in the two age groups. Similar to the trend seen in C57 mice, downregulated miRNAs eclipsed upregulated miRNAs by a ratio of approximately 7.5∶1 (52/7). This ratio was substantially greater than that seen for C57 mice.

**Figure 5:**
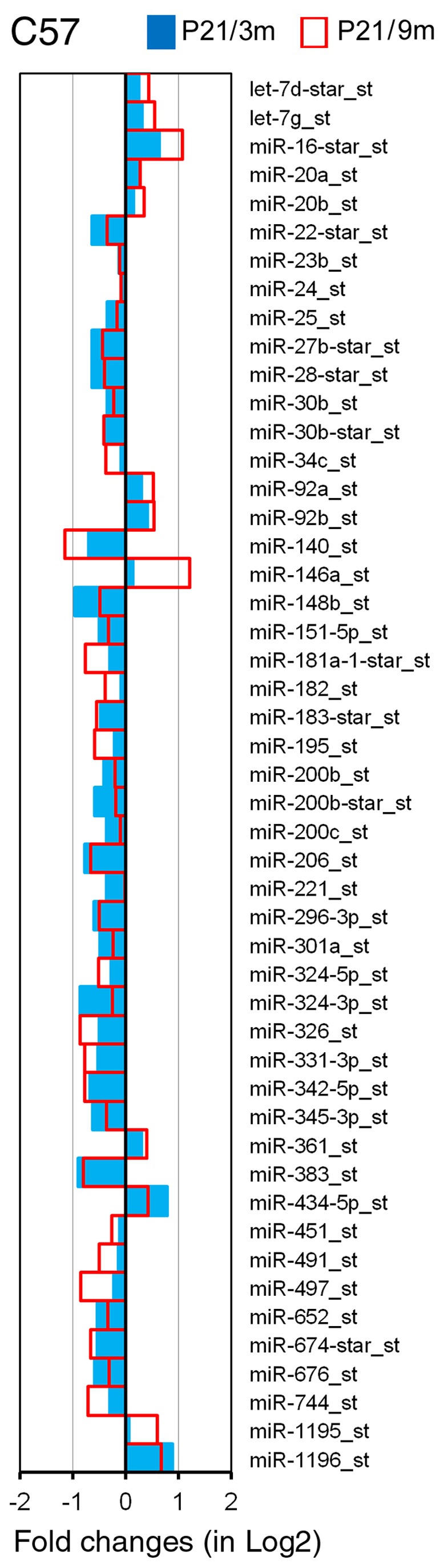
miRNAs that are uniquely expressed in the OC of C57 mice during aging. The X-axis relates Log2 transformed fold change in miRNA expression 3 m (in blue) and 9 m (in red) compared to P21.

C57 and CBA mice have different genetic backgrounds, which might contribute to different miRNA expression. The early onset and rapid progression of hearing loss seen in C57 mice is due to *Ahl* gene mutation [27], [28], 35. Thus, some differences in miRNA expression are likely associated with the *Ahl* mutation that may cause early degeneration of stereocilia and the mechanotranduction apparatus, leading to hair cell apoptosis. We thus examined and compared the miRNA expression profile of C57 mice with that of CBA mice. Forty-nine miRNAs were found to be differentially expressed only in C57 mice (Fig. 5). Among the list, twelve miRNAs were significantly up-regulated, while thirty-seven miRNAs were significantly down-regulated.

**Figure 6:**
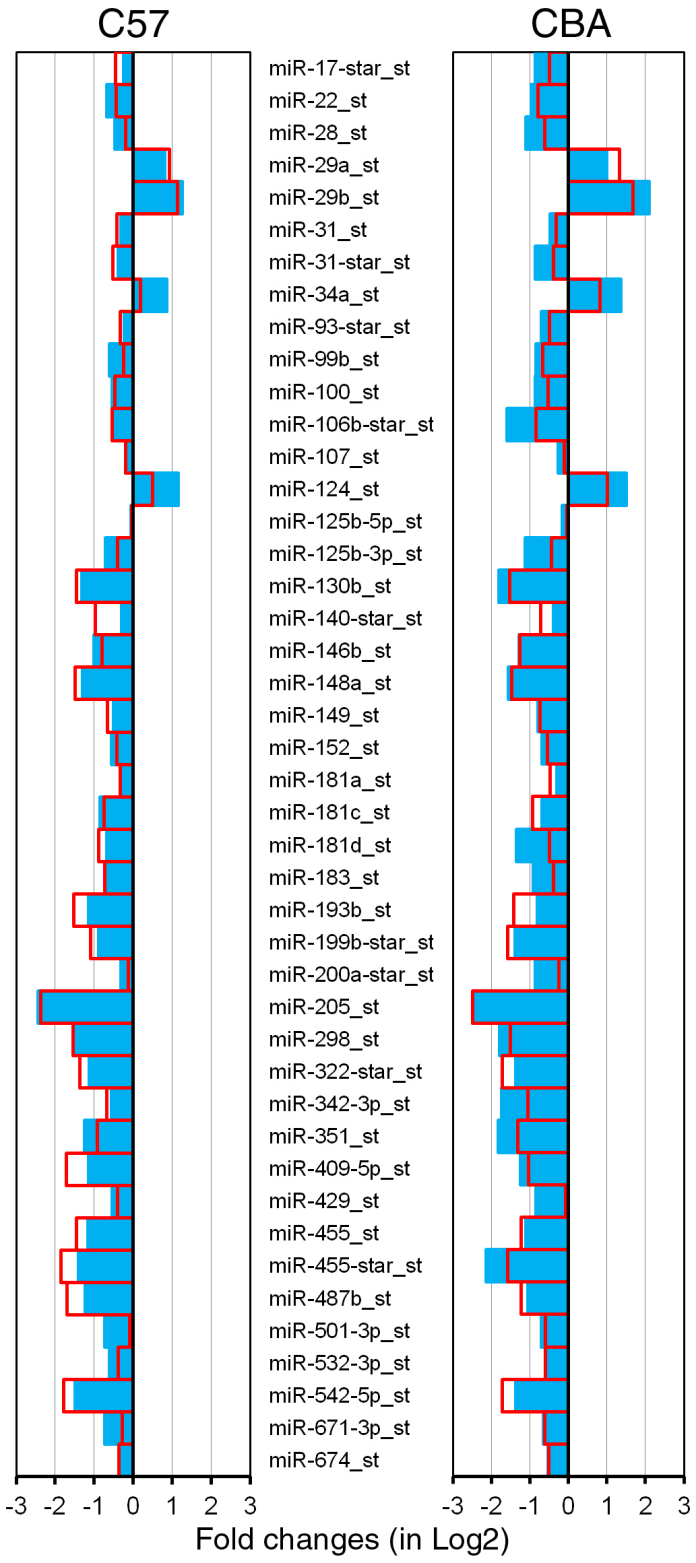
miRNAs that are commonly expressed in the OC of C57 and CBA mice during aging. The X-axes relate Log2 transformed fold change in miRNA expression for C57 mice at 3 m (in blue) and 9 m (in red) compared to P21 (left panel), and for CBA mice at 9 m (in blue) and 16 m (in red) compared to P21 (right panel).

Despite the differences between C57 and CBA mice, these two strains should share some common miRNAs that are associated with hair cell apoptosis and degeneration of the OC. To identify common miRNAs that were involved in ARHL in both strains, we also identified miRNAs that were present in both C57 and CBA mice. Figure 6 depicts 44 miRNAs that were present in both strains. Among this list, forty miRNAs showed significant down-regulation whereas four miRNAs, miR-29a, miR-29b, miR-34a and miR-124, were up-regulated.

### 4.Validation by q-PCR Analyses

**Figure 7:**
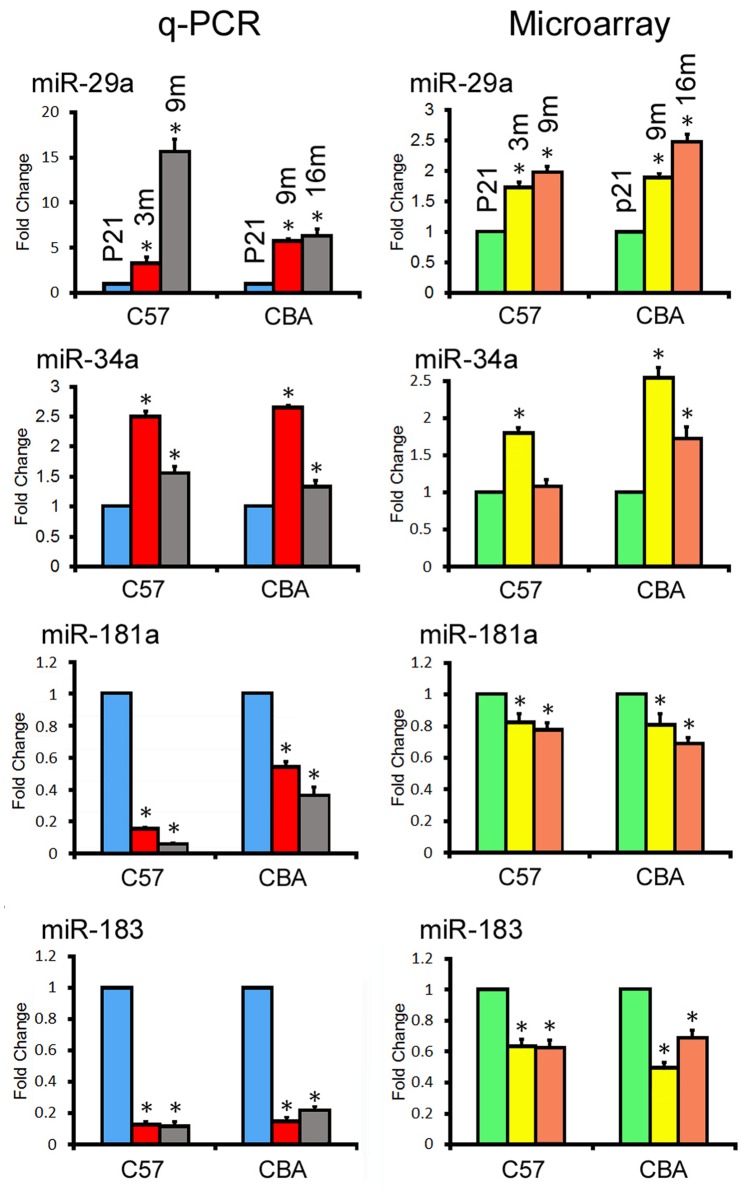
Comparison of changes in miRNA expression detected by q-PCR versus microarray analyses for four miRNAs in the OC of C57 and CBA mice. Asterisks indicate statistically significant differences (p<0.05, Student’s t-test) compared to P21. Each q-PCR plot represents means from three repeats.

We used q-PCR assays to validate differential expression of some of the miRNAs identified by microarray analyses. Two upregulated and two downregulated miRNAs were selected for q-PCR analyses. The two upregulated miRNAs, miR-29a and miR-34a, are known to regulate apoptosis pathways [36]–[38]. The two downregulated miRNAs, miR-181 and miR-183, are important for proliferation and differentiation, respectively [39]–[42]. The OC from the apical turn was selected for q-PCR analyses since the apical turn had minimal hair cell loss during aging, even for C57 mice. Using U6 RNA as an internal control, the change in miRNA expression was determined comparing older groups to P21. Figure 7 exhibits changes in expression of four different miRNAs during aging using q-PCR. For comparison, the change in expression of these four miRNAs from microarray analyses is also presented. As shown, the expression levels of miR-183 and miR-181 were significantly downregulated, while miR-29a and miR-34a were upregulated with aging compared to P21. These changes in expression are consistent with the trends seen in microarray analyses.

## Discussion

The analysis of miRNAs that are differentially expressed in tissues from younger and older organisms has led to the discovery of numerous miRNAs that are important for controlling aging processes in previous studies [5], [6], [8], [9], [43]. We used high-throughput microarray analysis to identify miRNAs that were up- or downregulated in the OC in two strains of mice during aging. We show that approximately 111 and 71 miRNAs exhibit differential expression in C57 and CBA mice. Many miRNAs indicated by our microarray analyses (Figures 3 and 4) were previously known to be involved in differentiation, proliferation, growth, and apoptosis in other tissues and organs [44]–[47]. The precise functions and specific targets of these miRNAs in regulating cellular senescence and aging are yet to be revealed. However, changes in expression levels during aging suggest that these miRNAs are directly and/or indirectly involved in regulatory processes that contribute to degeneration of the OC.

The majority of miRNAs identified in the present study can be classified into two categories by their perceived functions in the aging process: anti-apoptotic (or pro-growth) and pro-apoptotic. Most miRNAs that are significantly upregulated with aging in the OC are pro-apoptotic, while most downregulated miRNAs are pro-growth. The miRNAs in the pro-apoptotic category include miR-29a/b/c, miR-34a/b/c, let-7a/b/c/e/f/g/i, miR-141, miR-146, miR-203, miR-429, and several others. The anti-apoptotic category of miRNAs includes miR-17, miR-181a/b/d, and miR-182/183 [45]. These miRNAs can work through different pathways to directly or indirectly affect apoptosis through multiple transcript factors and functional proteins such as p53, the Bcl2 family, p27, the TNF receptors super family, caspases and caspase regulators. We summarize the known functions and regulatory mechanisms of these anti- and pro-apoptotic miRNAs in Table S1.

miRNAs that showed approximately 2-fold upregulation include members of the miR-29 family and miR-34 family, which have been demonstrated to be involved in cellular senescence and apoptosis in cell lines, tissues, and organisms during aging [48]–[51]. Both miR-29 and miR-34 can affect genes that activate or enhance p53 pathways. Therefore, these two miRNAs are generally considered to be pro-apoptotic [48], [49]. miR-29 can suppress several important genes that drive cell survival (e.g. Cdc42, p85a, Mcl1, and Tcl1). miR-29 family members indirectly upregulate p53 levels and induce apoptosis in a p53-dependent manner by suppressing p85a (the regulatory subunit of PI3Kinase) and CDC42 (a Rho family GTPase), both of which negatively regulate p53 [52]. Studies have also shown that miR-29a is able to revert DNA methylation by targeting DNA methyltransferases 3A (Dnmt 3a) and 3B (Dnmt3b) [53]. Overexpression of miR-29 family members inhibits cell proliferation [50]. High levels of miR-29 may render the cells more susceptible to p53-dependent stress responses [54]. miR-29a and miR-29b were significantly upregulated in both strains of mice in our study. The increased expression of miR-29 during aging and the pro-apoptotic nature of this family indicate miR-29 is likely involved in the degeneration of the OC.

Another p53-relevant miRNA indicated in the process of ARHL in our study is miR-34a. miR-34 family members participate in downstream signaling of the p53 pathway [55]. The level of miR-34a expression may affect the decision between apoptosis and cell-cycle arrest. Studies have shown that p53 can bind directly to the promoter and activate miR-34 genes in response to DNA damage and oncogenic stress. miR-34 mediates the downstream effects of p53 by suppressing a number of genes including CDK4/6, Cyclin E2, MET, and Bcl-2, thereby promoting apoptosis [56], [57]. One recent study showed that miR-34a expression in the inner ear was drastically upregulated during the course of damage induced by ototoxic drugs [58]. In the present study, the highest expression level of miR-34a appeared amid the progression of ARHL. Whereas miR-29 regulates genes upstream of p53 pathways and miR-34a regulates genes downstream of p53 pathways, the results suggest that miRNAs contribute to p53-dependent apoptosis in degeneration of the OC in the inner ear. The role of p53 in hair cell apoptosis and ARHL has been demonstrated in a number of studies [59]–[64].

miRNAs that were significantly downregulated include members of the miR-181 and miR-183 families. The miR-181 family is known to mediate proliferation in many cells [54], [65], [66]. Previous studies have shown that transfection of miR-181b in HeLa and HCT-116 tumor cells regulates a large number of genes, inducing those related to cell growth. miR-181a has been specifically shown to have a proliferative effect in human myeloid leukemia cells [67]. This effect appears to be mediated in part by downregulation of the p27 pathway [39]. In the inner ear, miR-181a is upregulated during regeneration of the sensory epithelium in the basilar papilla of chicken, suggesting that miR-181a is involved in mechanisms that control proliferation and differentiation in the auditory sensory epithelium [19]. Among all the family members, miR-181a/b/d exhibited significant downregulation in the OC of both strains in our microarray analyses and q-PCR validation.

miR-183 family members include miR-96, miR-182, and miR-183. This family regulates genes that are associated with differentiation, proliferation, and growth in various types of normal and cancer cells [68], [69]. The family is abundantly expressed in sensory neurons and hair cells into adulthood in the inner ear, and it is important for cell specification and hair cell fate determination [12]. Recent studies show that two single-base mutations in the seed region of miR-96 result in autosomal dominant, progressive hearing loss in both humans and mice [70], [71]. The mutation alters the function of miR-96 and gene expression profiles in mouse OC. Moreover, hair cell gene expression required for normal function is considerably perturbed. Five genes, *Ocm* [72], *Pitpnm1* [73], *prestin* [74], *Ptprq* [75], and *Gfi1* [76], are among disregulated genes in the mutant mice. All of these genes are specifically expressed in hair cells, and mutations in each of the latter three genes are known to result in deafness and hair cell degeneration [77]–[79]. We observed a significant downregulation of miR-182 and miR-183 in the two strains. Interestingly, miR-96 downregulation was only detected in C57 mice at 9 m. No significant change of miR-96 was detected in CBA and C57 mice at 3 m. The fact that miR-96 did not show a significant change in the early stage of degeneration of the OC suggests that significant change of miR-96 might only occur during the later stage of ARHL.

Weston et al. [12] examined miRNA expression in the mouse inner ear during development between P0 and P100 by microarray analyses. Their study uncovered 76 miRNAs expressed in the whole inner ear including ganglion neurons, the basilar membrane, and the cochlear lateral wall. The present study identified more miRNAs despite the fact that the tissue collected only contained the basilar membrane containing the OC. Comparison of the two studies shows that most miRNAs indicated in the miRNA expression profile of Weston et al. [12] are also present in our study. The majority of miRNAs that are present in their profile and absent in our analysis are those important for proliferation and differentiation. These miRNAs include miR-135, miR-143/145, miR-189, and miR-204. One miRNA worthy of mention is the miR-143/145 cluster. This cluster has been shown to be involved in cardiac morphogenesis and smooth muscle fate determination [80]. The miR-143/145 cluster has also been implicated in tumorgenesis of various types of carcinoma by suppressing the RAS, c-Myc, and human telomerase (hTERT) signaling pathways [81], [82]. We note that most miRNAs that are not in the miRNA profile in the study by Weston et al. [12] are miRNAs that were not available in the oligonucleotide microarray in 2006. These miRNAs include miR-652, miR-711, miR-744, and miR-762, all of which are downregulated during aging. The functions and regulatory mechanisms of these miRNAs in the auditory system are not known. However, recent studies have revealed their involvement in age-related diseases, such as heart disease, hypertension, and age-related metabolic syndrome [83]. For example, miR-652 is upregulated, whereas miR-744 is downregulated in older hearts. miR-744 is also found differentially expressed in senescent cell lines and mesenchymal stem cells. Short-term overexpression of miR-744 results in enhanced cell proliferation, while long-term expression causes chromosomal instability and tumor suppression *in vivo* [84].

One of the important findings of our study is that the majority of expressed miRNAs decline in relative abundance during aging. Downregulated miRNAs outnumbered upregulated miRNAs by a wide margin. This global decline of miRNA during aging is consistent with the trend observed in other studies [85]. For example, in the aging brain, 85 miRNAs are downregulated while only 8 miRNAs are upregulated [85]. The vast majority of *C. elegans* miRNAs that are differentially expressed during aging are also downregulated [7]. Interestingly, the majority of miRNAs that are in decline are known regulators for differentiation, proliferation, and growth, whereas those miRNAs that are upregulated are all known pro-apoptotic regulators. Our study likewise suggests that age-related degeneration of the OC is a complex process that involves a shift in the balance of pro-growth and differentiation miRNAs and factors toward pro-apoptotic miRNAs and factors.

Hair cell loss was observed initially in the basal end of the OC during aging; hair cell loss occurred as early as 3 months in C57 mice. Loss of hair cells and supporting cells can lead to global decline of miRNAs in the older tissue when compared to the younger tissue. However, it is unlikely that loss of hair cells and supporting cells is the reason behind the decline of some miRNAs in the older tissue for the following reasons: first, changes in miRNA expression were observed even before hair cell loss occurred. In CBA mice, hair cell loss did not occur until 16 months, while downregulation of many miRNAs was already observed at 9 months (blue color in Fig. 4); second, although there was some hair cell loss at 3 months (in C57 mice) and 16 months (in CBA mice), the loss was limited to the basal region and accounted for less than 10% of the hair cells (Fig. 2). This limited reduction would translate to less than 2–3% of total hair cell populations in the whole cochlea. Such nominal loss of hair cells is unlikely to account for the significant decline of miRNAs observed in our microarray analyses. Finally, we validated four different types of miRNAs using q-PCR. To avoid the issue of hair cell loss, the tissue was collected from the apical turn where hair cell loss was insignificant at all ages (top panels in Fig. 2C,D). Our q-PCR analyses were consistent with the expression patterns observed in microarray analyses. Taken together, we conclude that it is unlikely that hair cell loss is responsible for reduced expression of the majority of miRNAs observed in the aging cochlea.

We observed substantial differences in the number and amount of miRNA expression between C57 and CBA mice. The extent and amount of miRNA changes depend on the molecular mechanisms and the stage of aging. Different genetic backgrounds of the two strains may also contribute to different miRNA expression. The early onset and rapid progression of hearing loss seen in C57 mice is due to *Ahl* gene mutation [27], [28], [35]. Thus, some differences in miRNA expression are likely associated with the *Ahl* mutation.

We examined miRNA expression at different stages during aging for the two strains of mice. These different time points represent different phases before and after the onset of ARHL. For C57 mice, high frequency hearing loss accompanied by some hair cell loss in the basal end of the cochlea already occurred at 3 months. Thus, comparison of miRNA expression at P21 with those at 3 m and 9 m reveals miRNAs involved in progression of ARHL. For CBA mice, the hearing function was normal and no significant hair cell loss was detected at 9 months. However, significant reduction of pro-growth miRNAs and upregulation of pro-apoptotic miRNAs was already detectible at this age. This suggests that changes in miRNA expression precede morphological and functional changes. The fact that the shift from pro-growth to pro-apoptotic processes starts well before the onset of ARHL suggests that miRNAs are contributing factors to the onset and progress of ARHL.

In conclusion, our study for the first time demonstrates the extent and specificity of miRNA expression during aging in the mammalian inner ear. We show that the underlying process and regulatory mechanism of aging in the auditory sensory epithelium involve repression of miRNAs important for proliferation and differentiation and enhancement of miRNAs that promote apoptosis. Such change in the miRNA expression profile takes place well before morphological changes and hearing loss are detected. The present work is the first step in an effort to elucidate the roles of miRNAs and their regulatory networks in age-related degeneration of the OC. It also lays the groundwork for future experiments that can explore whether suppression or overexpression of some miRNAs can slow the onset and progression of ARHL.

## Supporting Information

Table S1 **Known mechanisms of some anti- and pro-apoptotic miRNAs identified in the OC during aging.**
(PDF)
