# Supplementary material for: Identifying MicroRNAs Involved in Degeneration of the Organ of Corti during Age-Related Hearing Loss
Source: PLoS One. 2013 Apr 30;8(4):e62786. doi: 10.1371/journal.pone.0062786 (PMC3640032; doi:10.1371/journal.pone.0062786)
Supplement: Table S1 — Known mechanisms of some anti- and pro-apoptotic miRNAs identified in the OC during aging. (PDF) [file pone.0062786.s001.pdf]

Table 1. Known mechanisms of some anti- and pro-apoptotic miRNAs identified in the OC during aging

Probe sets (miR family)	Expression pattern (with aging)	Potential target genes and pathways (aging relevant)
miR-15b	Down-regulated	Directly targeting mitogenactivated protein kinase kinase (MKK4), targeting on Bcl2 protein in gastric cancer cells, CLL patients, and targeting on cyclin E1 in glioma cells.
miR-24	Down-regulated	Inhibits Cell Proliferation by targeting E2F2, MYC, p16INK4a, MKK4 and H2AX, contributing to the senescence and to overcome the additional oxidative stress and DNA damage with aging. Negatively regulates the pro-apoptotic factors caspase9 and apaf1, regulating apoptosis during normal development (in neural retina).
miR-28-3p/miR-28-5p	Down-regulated	Targeting 3'UTR of NF-E2-related factor 2 (Nrf2) in breast epithelial cells, involved in antioxidant response. Target 3'-UTR of alternative splicing factor/splicing factor 2 (ASF/SF2) in mouse embryo fibroblasts (MEF), and induces apoptosis and senescence.
miR-29a	Up-regulated	Target on negatively regulating factors of p53: CDC42 and P85a subunit, and involved in DNA damage apoptosis pathway.
miR-99b /miR-100	Down-regulated	Tumor-suppressive miRNAs, targeting on chromatin-remodeling factors SMARCA5, SMARCD1 and the growth regulatory kinase mTOR in prostate cancer cells and breast cancer cells.
miR-130b	Down-regulated	Associated with stress induced premature senescence and replicative senescence in human primary keratinocytes. Targeting on tumor suppressor gene TP53INP1 and p63. Directly targeting the 3-UTR of p21Cip1, promoting gastric cancer by down-regulating the tumor suppressor, runt-related transcription factor 3 (RUNX3).
miR-141	Up-regulated	EMT inhibitors, negatively regulating ZEB1/ZEB2 pathway. Directly targeting mitogenactivated protein kinase kinase (MKK4).
miR-146b	Down-regulated	Components of the MAPK pathway, negatively regulating the senescence-associated secretion of IL-6 and IL-8 by directly targeting IRAK1, TRAF6; down-regulating the SASP by reducing NF-κB activity.

miR-148a	Down-regulated	Directly inhibited DNMT1 expression by targeting the protein coding region of its transcript, contributing to DNA Hypomethylation in Lupus CD4+ T Cells. Targets 3□ UTR of rapamycin-insensitive companion of mTOR which is integral to growth and proliferation, extending lifespan.
miR-181a/d	Down-regulated	Mediate of proliferation, regulates Bcl2, p27, and associated with replicative senescence in human primary keratinocytes.
miR-182/miR-183	Down-regulated	A potential oncomir family through regulating two tumor suppressor genes, EGR1 and PTEN in many tumor types. MiR-183 appears to be a transcriptional target of p53 and targeting the expression of ZEB1, TCF-4, and NFKB. MiR-182 contributes to specific changes in gene expression associated with senescence and aging, such as SIPS, RARG and FOXO3.<br>Strongly and specifically expressed in hair cells. Repress either prosensory genes or supporting cell genes to help specify hair cell fate, (such as Sox2). Targeting on Integrin beta 1 and Kinesin 2 alpha contributes to functional alterations associated with senescence in neurosensory organs.
miR-199b/miR-199b*	Down-regulated	MiR-199b* is a regulator of the Notch pathway by targeting on HES1whose down-regulation regulates the proliferation rate and anchorage-independent growth of MB cells. MiR-199b targets the nuclear NFAT kinase dual-specificity tyrosine-(Y)-phosphorylation regulated kinase 1a (Dyrk1a) in mouse and human heart failure.
miR-298	Down-regulated	Up-regulated in most of the cancer cell lines and involved in carcinogenesis. Targets 3'-UTR of BACE1 mRNA in Alzheimer's Disease, leading to increased Aβ formation and disease progression.
miR-326	Down-regulated	Predicted targets includes vitamin D receptor and Erythroblastosis virus E26 oncogene homologue, expressed at higher levels in T1diabetes and multiple sclerosis subjects with ongoing islet autoimmunity. Modulates expression of multidrug resistance-associated protein 1 in chemotherapy resistance of breast cancer.
miR-338-5p	Down-regulated	A family of brain-specific microRNA precursors found in mammals and humans. It is sufficient to promote oligodendrocyte differentiation, repressing Sox6 and Hes5. Putative targets include a number of transcription regulators of apoptosis-associated tyrosine kinase (AATK).
miR-342-3p	Down-regulated	Primarily brain and spleen- specific functioning as a pro-apoptotic tumor suppressor. Commonly suppressed in human colorectal cancer, breast cancer and melanoma by means of hypermethylation of the Ena/Vasp-like CpG island, and its restoration induces an apoptotic response.

miR-351	Down-regulated	Target important factors during apoptosis, including TNF-α, death activator FasL, effector caspases including caspases 3 and 7, pro-apoptotic members such as Bax, p53, and NF-κB; and anti-apoptotic members such as IRAK-2 and BCL2. Negatively regulates TMEM59 expression in different cell types.
miR-409-5p	Down-regulated	known as brain-enriched or even brain-specific miRNAs.
miR-423-3p	Down-regulated	Target 3' UTR of tumor suppressor p21Cip1/Waf1. Significantly promotes cell proliferation and cell cycle progression at the G(1)/S transition in hepatocellular carcinoma cells.
miR-433	Down-regulated	Negatively regulates hematopoietic cell proliferation and erythropoiesis, and down-regulated in gastric carcinoma by directly targeting tumor-associated proteins GBP2. Target the glucocorticoid receptor: Nr3c1, MAD2and HDAC6 in different cell types. Target on FGF20 which correlates with increased alfa-synuclein expression in Parkinson patients.
miR-455/ miR-455*	Down-regulated	Host gene: Col27a1, which encodes a cartilage collagen, regulates apoptosis in adult articular cartilage and muscle tissues. Has 3 direct targets: Smad2/3, activin receptor 2B, and chordin-like 1. Induced by TGF1, TGF3, and activin. Predicted to target on PAX6, which plays a critical role in the self-renewal and differentiation of neural stem cells.
miR-467a	Down-regulated	Directly target on key apoptotic mediators: Caspase2 and Ei24 (a p53 transcriptional target) in mouse embryonic stem cells.
miR-676<br>miR-125b-2p	Down-regulated	MiR-125b-3p and miR-676 are directly or indirectly regulated by the level of functional p53. MiR-125b has been identified as both a target of p53 and a regulator of the p53 transcript itself, inhibiting neuroblastoma cell proliferation in vitro.
